# Iron Competition as an Important Mechanism of Pulcherrimin-Producing *Metschnikowia* sp. Strains for Controlling Postharvest Fungal Decays on Citrus Fruit

**DOI:** 10.3390/foods12234249

**Published:** 2023-11-24

**Authors:** Shupei Wang, Zhimei Tan, Chenshu Wang, Wenqing Liu, Fangxue Hang, Xuemei He, Dongqing Ye, Li Li, Jian Sun

**Affiliations:** 1 Guangxi Academy of Agricultural Sciences, Nanning 530007, China; wshp2000@163.com (S.W.); xuemeihe1981@126.com (X.H.); yedongqing@gxaas.net (D.Y.); lili@gxaas.net (L.L.); 2 Guangxi Key Laboratory of Fruits and Vegetables Storage-Processing Technology, Nanning 530007, China; 3 College of Environmental and Life Sciences, Nanning Normal University, Nanning 530001, China; 4 College of Light Industry and Food Engineering, Guangxi University, Nanning 530004, China; tzhm0810@163.com (Z.T.); cswang0321@163.com (C.W.); wqliue@163.com (W.L.); hangfx@163.com (F.H.)

**Keywords:** *Metschnikowia pulcherrima*, pulcherrimin, iron competition, *P. digitatum*, *P. italicum*, *G. citri-aurantii*

## Abstract

This study identified and tested fruit-isolated *Metschnikowia* yeasts against three major postharvest citrus pathogens, namely, *Penicillium digitatum*, *Penicillium italicum*, and *Geotrichum citri-aurantii*, and further evaluated the impact of FeCl_3_ on the biocontrol efficiency of pulcherrimin-producing *M. pulcherrima* strains. Based on the characterization of the pigmented halo surrounding the colonies and the analysis of the D1/D2 domain of 26S rDNA, a total of 46 *Metschnikowia* sp. were screened and identified. All 46 *Metschnikowia* strains significantly inhibited the hyphal growth of *Penicillium digitatum*, *Penicillium italicum*, and *Geotrichum citri-aurantii*, and effectively controlled the development of green mold, blue mold and sour rot of citrus fruit. The introduction of exogenous FeCl_3_ at certain concentrations did not significantly impact the pulcherriminic acid (PA) production of pigmented *M. pulcherrima* strains, but notably diminished the size of pigmented zones and the biocontrol efficacy against the three pathogens. Iron deficiency sensitivity experiments revealed that *P. digitatum* and *P. italicum* exhibited higher sensitivity compared to *G. citri-aurantii*, indicating that iron dependence varied among the three pathogens. These results suggested that *M. pulcherrima* strains, capable of producing high yields of PA, possessed great potential for use as biocontrol agents against postharvest citrus diseases. The biocontrol efficacy of these yeasts is mainly attributed to their ability to competitively deplete iron ions in a shared environment, with the magnitude of their pigmented halo directly correlating to their antagonistic capability. It is worth noting that the level of sensitivity of pathogens to iron deficiency might also affect the biocontrol effect of pulcherrimin-producing *M. pulcherrima*.

## 1. Introduction

As one of the most commercially valuable fruits, citrus (*Citrus* spp.) is highly appreciated by consumers due to its unique flavor and rich nutrients [1]. Citrus fruits, however, are highly susceptible to spoilage fungi, including *Penicillium digitatum* (green mold), *Penicillium italicum* (blue mold), and *Geotrichum citri-aurantii* (sour rot) during postharvest, causing substantial economic losses [2]. With rising public concerns about the effects of synthetic chemical fungicides on food safety and environmental preservation, the pursuit of eco-friendly management techniques is intensifying. One of the most promising methods employs antagonistic yeasts as biological controls against postharvest decay in various fruits [3,4].

*Metschnikowia pulcherrima* is a ubiquitous yeast that exhibits strong ecological adaptability and is widely distributed across the globe. It is commonly found in flowers, nectars, and fresh and spoiled fruits [5]. This yeast typically produces pulcherrimin, a red pigment, which results in distinctive colony features [6]. *M. pulcherrima* possesses excellent antifungal properties. This was demonstrated by conducting competition assays. These involved using a sample of 40 yeasts on a diverse collection of 16 filamentous fungi, revealing *M. pulcherrima* to be the most potent antagonist [7]. Furthermore, when applied to freshly cut fruits, *M. pulcherrima* effectively inhibited the growth and proliferation of foodborne pathogens, including *Listeria monocytogenes* and *Salmonella enterica* [8,9]. In terms of controlling postharvest diseases, fruit-borne strains of *M. pulcherrima* exhibit strong antagonistic activity against a range of pathogens including *Penicillium* spp., *Botrytis cinerea*, *Alternaria alternata, Fusarium* spp., *Monilinia* spp., and *Pestalotiopsis vismiae*. Research has shown these strains to be highly effective in managing postharvest diseases of some fruits, such as apples, pears, sweet cherries, strawberries, lemons, grapes, loquats, and mangoes [10,11,12,13,14,15,16]. *M. pulcherrima* strains demonstrate the traits of an “ideal antagonist” because of their capacity to withstand harsh environmental circumstances, effectively outcompete for nutrients and space, and refrain from generating toxic substances [17,18]. These attributes, combined with its broad-spectrum antifungal qualities and biosafety, make *M. pulcherrima* a promising candidate for commercial application.

Iron (Fe) is an essential micronutrient for the survival and growth of almost all living organisms on earth. Competition for or depletion of iron ions are considered important mechanisms of action in the biocontrol of postharvest fruit diseases by pulcherrimin-producing *Metschnikowia* strains, including *M. pulcherrima*, *M.* andauensis, *M. fructicola*, *M. citriensis*, etc. [17,19]. These yeasts compete for iron ions with pathogens through the secretion of a siderophore—pulcherriminic acid (PA). This water-soluble PA is capable of spontaneously chelating four Fe^3+^ ions in a non-enzymatic manner, resulting in the formation of the red, insoluble pulcherrimin complex [20]. This process results in iron fixation in the coexistence environment, thereby depleting the availability of free iron and further inhibiting the growth of various bacteria and pathogenic fungi [6,17,21]. Therefore, pulcherrimin-producing *Metschnikowia* strains are regarded as highly promising for biocontrol agents [22].

Limited information suggests that *M. pulcherrima* has the potential to control green mold in lemons and mandarins [16,23]. However, there is little information available about the effectiveness of *M. pulcherrima* against citrus blue mold and sour rot. The aim of this research was to assess the biocontrol efficacy of fruit-sourced *Metschnikowia* yeasts against three postharvest citrus pathogens, and further to investigate the competitive ability of *M. pulcherrima* for iron acquisition against *P. digitatum*, *P. italicum*, and *G. citri-aurantii*. This study could provide new high-performance antagonistic yeast resources for the biocontrol of postharvest citrus diseases, as well as a certain theoretical basis for the investigation of iron competition mechanisms of *M. pulcherrima*.

## 2. Materials and Methods

### 2.1. Fruit Material

Commercially mature Olinda Valencia and Newhall navel oranges [*Citrus sinensis* (L.) Osbeck] were harvested from the commercial orchard located in Hepu and Fuchuan country, Guangxi, China, respectively. The selected fruits, which displayed consistent size, shape, and ripeness and showed no visible symptoms of disease or mechanical injury, were surface-sterilized via dipping in a 2% (*v*/*v*) sodium hypochlorite solution for 2 min. They were then thoroughly washed with tap water and left to air-dry at room temperature for the next experiment.

### 2.2. Pathogens Preparation

The strains of *P. digitatum*, *P. italicum* and *G. citri-aurantii* were identified, isolated from naturally decayed citrus fruits exhibiting disease-related symptoms and preserved in our laboratory. Before use, all isolates were cultivated on potato dextrose agar (PDA) at 25 °C for 5 days, and then the spores of the three pathogens were rinsed off with sterile water. The resulting cell numbers were calculated using a hemocytometer and adjusted to the desired concentrations.

### 2.3. Sampling and Yeast Isolation

The yeasts of the genus *Metschnikowia* were isolated from the different cultivars of table grapes collected in turn at 15 d intervals from agricultural wholesale markets in Xixiangtang (XX), Jiangnan (JN), Xingning (XN) and Wuming (WM) districts of Nanning, Guangxi, respectively, from April to October 2021. After labeling, the samples were stored in sterile self-sealing bags and transported back to the laboratory aseptically. Grapes were picked and rinsed four times with sterile water. Approximately 30–50 grapes from each sample were then randomly selected and incubated in a triangular flask containing 500 mL of sterile water at 28 °C for 3 h with shaking (180 rpm). The yeast suspension was then diluted 10-fold, and 50 μL of the dilution was plated on YPD + Fe plates (20 g L^−1^ peptone, 20 g L^−1^ dextrose, 10 g L^−1^ yeast extract, 20 g L^−1^ agar, 5 mg L^−1^ FeCl_3_) containing 50 mg L^−1^ of kanamycin. All the media formulations were obtained from Solaibao Biological Technology Co., Ltd. (Beijing, China). After culturing at 28 °C for 48 h, the reddish colonies were picked based on pulcherrimin pigment production characteristics and investigated for cell morphology using an optical microscope. Afterwards, the single colonies were purified via re-streaking on YPD + Fe plates three times and the pure yeast cultures were subsequently stored in glycerol stocks at −80 °C.

### 2.4. Yeast Identification

The isolates were grown in YEPD medium (YPD without agar) at 28 °C (180 rpm) to reach the mid-log phase, after which the cells were collected for genomic DNA extraction using an Ezup column yeast genomic DNA purification kit according to the manufacturer’s protocol (Sangon Biotech, Shanghai, China). The D1/D2 region of the 26S rDNA of the yeast isolates was amplified using the universal primers NL1 (5′-GCATATCAATAAGCGGAGGAAAAG-3′) and NL4 (5′-GGTCCGTGTTTCAAGACGG-3′), as previously described [24]. The PCR products were sequenced by Sanger sequencing (Sangon Biotech Co., Ltd., Shanghai, China), and the resulting sequences were blasted against the NCBI nucleotide database (http://blast.ncbi.nlm.nih.gov/, accessed on 11 December 2022) with BLASTn for species identification.

### 2.5. Antagonism Assays

#### 2.5.1. In Vitro Biocontrol Assay on Agar Plates

The inhibitory effects of the *Metschnikowia* yeasts against *P. digitatum*, *P. italicum*, and *G. citri-aurantii* were tested on PDA plates, using the method of Wang et al. [25] with slight modifications. The yeast isolate cells (20 μL, 1 × 10^8^ cells mL^−1^) were streaked evenly along the central axis of a PDA plate, covering an area of 60 mm long × 3 mm wide. After incubation for 10 min, 5 μL conidial suspensions (1 × 10^6^ spores mL^−1^) of *P. digitatum*, *P. italicum* and *G. citri-aurantii* were inoculated at 30 mm on both sides of the coating position. The width of the inhibition zone (subtract the width of the yeast growth zone) was measured after 7 d of incubation at 25 °C. Three replicates of each treatment were performed, and each replicate contained six plates.

In addition, five strains of *M. pulcherrima* were used as controls: *M. pulcherrima* (CICC 33433, CICC 1467, CICC 32343, and CICC 33447) was purchased from the China Center of Industrial Culture Collection (CICC), and *M. pulcherrima* (CGMCC 2.3314) was obtained from the China General Microbiological Culture Collection Center (CGMCC). These five yeast strains were activated via inoculation in YPD medium for 48 h before use.

#### 2.5.2. In Vivo Biocontrol Assay on Citrus Fruits

All yeast isolates were evaluated for biocontrol activity against the three major post-harvest citrus diseases, following the method described by Liu et al. [26] with some modifications. The yeast strains were inoculated in 100 mL YEPD medium and incubated at 28 °C and 200 rpm for 16 h to reach the mid-log phase, and then centrifuged at 8000× *g* for 5 min to collect the cells. After being washed twice with sterile water, the concentration of yeast cells was counted and adjusted to 1 × 10^8^ cells mL^−1^ for each treatment group. The prepared citrus fruits were artificially wounded (approximately 3 mm diameter and 3 mm deep) on opposite sides of their equators with a sterile 1000 μL pipette tip. Each wound was then inoculated with 20 μL of yeast cell suspension (1 × 10^8^ cells mL^−1^), and an equal amount of sterile water was used as a control. After 4 h, 10 μL spore suspensions of *P. digitatum* (1 × 10^4^ spores mL^−l^), *P. italicum* (1 × 10^4^ spores mL^−l^) and *G. citri-aurantii* (1 × 10^6^ spores mL^−1^) were added to each wound. After being air-dried, all treated fruits were individually wrapped in high-density polyethylene plastic bags and stored at 25 ± 2 °C with 85–90% relative humidity (RH). The disease incidence (DI) and lesion diameter (LD) were counted daily at 1 d intervals after the appearance of disease characteristics on the fruit. Each treatment was replicated three times, with each replicate consisting of 10 fruits.

### 2.6. Test for Pulcherrimin Pigment Production of Yeast Isolates

The pulcherrimin pigment produced by *Metschnikowia* yeasts was tested using PDA + Fe plates with a concentration of 10 mg L^−1^ FeCl_3_. In total, 10 mL of molten medium was poured into Petri dishes and allowed to set. Next, a 20 μL yeast cells suspension (1 × 10^8^ cells mL^−1^) was evenly spread along the plate’s central axis, covering an area of 60 mm in length and 30 mm in width. The width of the pigmented halos surrounding the yeast colonies was recorded following a 3-day incubation period at 28 °C. There were six replicate plates for each yeast isolate, and the experiment was reproduced twice.

### 2.7. Iron Competition and Antagonism

#### 2.7.1. Iron Competition Assay on Agar Plates In Vitro

The effect of iron on the growth of pulcherrimin-producing *Metschnikowia* yeasts, inhibiting *P. digitatum*, *P. italicum*, and *G. citri-aurantii*, was tested using PDA plates with varying FeCl_3_ concentrations (0, 5, 10, 15, 20, 25, 50 mg L^−1^). This technique was referring to our previously published methods [27] with slight modifications. Overall, 10 mL PDA + Fe medium was poured into Petri plates and allowed to solidify; then, 20 μL of yeast cell suspension (1 × 10^8^ cells mL^−1^) was uniformly streaked along the plate’s central axis, covering an area measuring 60 × 30 mm (length × width). After 10 min, the conidia suspensions (5 μL, 1 × 10^6^ spores mL^−1^) of *P. digitatum*, *P. italicum* and *G. citri-aurantii* were positioned at 30 mm on either side of the yeast strip location. The width of the inhibition zone was measured after incubation for 7 d at 25 °C. In addition, the PDA plates coated with yeast alone were placed in a 28 °C incubator for 3 days; then, the width of the pigmented circles was measured. Five plates were prepared for each treatment, and the experiments were performed twice.

#### 2.7.2. Iron Competition on Citrus Fruits In Vivo

Citrus fruits with two artificial wounds were prepared as described in Section 2.5.2. The wounds were injected with 20 µL of (1) yeast cells suspension (1 × 10^8^ cells mL^−1^) and (2) sterile water as a control. After 4 h, 10 µL of spore suspensions of *P. digitatum* or *P. italicum* (1 × 10^4^ spores mL^−l^), and *G. citri-aurantii* (1 × 10^6^ spores mL^−1^) were applied to each wound. After drying, aliquots of 20 µL of FeCl_3_ (0, 5, 15, 25 mg L^−1^) were applied to each wound. After being bagged with the polyethylene bag, all fruits were stored at conditions of 25 °C and RH 85–90%. The DI and LD of each fruit was measured when the fruit showed symptoms of disease every 24 h. Each treatment was replicated 3 times with 10 fruits per repetition.

#### 2.7.3. Test Iron on the Growth and PA Production of Selected Yeasts

The PDB (potato dextrose broth), supplemented with FeCl_3_ at varying concentrations (0, 1.0, 5, 10, 15, 25, 50, 100 mg L^−1^), was employed to test the effect of iron on the cell growth and intracellular PA production of *Metschnikowia* yeasts. The selected yeasts were incubated at 28 °C for 16 h with 180 rpm shaking and harvested at the mid-log phase. After washing and counting, the yeast cells were seeded into 50 mL of PDB + Fe medium at a final concentration of 1 × 10^6^ cells mL^−1^, and cultured for 30 h in a shaking incubator (180 rpm) at 28 °C. The cell growth and PA production of the yeasts was determined by reference to the previous study [22,28,29]. Briefly, 6 mL fermentation broth was taken and centrifuged at 8000× *g* for 10 min at 4 °C; then, the precipitated cells were washed twice with sterile water and resuspended in 0.1 mmol L^−1^ NaOH to dissolve the pulcherrimin. Following centrifugation at 10,000× *g* for 2 min, the pellet was resuspended in sterile water. Subsequently, the yeast growth was evaluated by measuring optical density at OD_600_. Another 6 mL of yeast fermentation broth was harvested and centrifuged at 8000× *g* for 10 min. The resulting cell pellets were washed twice with deionized water and then resuspend in 2 mol L^−1^ NaOH to dissolve the PA. Subsequently, a centrifugation step (10,000× *g*, 3 min) was performed. The supernatant was then collected and the OD_410_ was measured using a UV-vis spectrophotometer (Tecan Infinite F-Plex plate reader) to determine the PA concentration. Each treatment was conducted in triplicate and each replicate was measured three times.

#### 2.7.4. Deficiency Sensitivity Tests for *P. digitatum*, *P. italicum* and *G. citri-aurantii*

The test pathogens’ sensitivity to iron deficiency was examined using tropolone (≥98%, Macklin, Shanghai, China), a chelating agent that exhibits a strong affinity for ferric ions [16]. An aliquot of 100 μL spore suspension of *P. digitatum*, *P. italicum*, and *G. citri-aurantii* at 1 × 10^6^ spores·mL^−1^ was uniformly spread on PDA plates. The plates were left to stand for 30 min until the spore suspension was fully adsorbed by the culture medium. Subsequently, a 5 mm diameter well in the center of the plate was produced via a sterilized punch. Then, 50 μL tropolone solution (5 mg mL^−1^) was added to the wells, and 50 μL sterile water was added as a control. After 3 days of incubation at 25 °C, the inhibition zone was measured. Six plates were prepared for each treatment, and the experiments were carried out twice.

### 2.8. Statistical Analysis

The statistical analysis was conducted utilizing SPSS 27.0 (SPSS Inc., Chicago, IL, USA). The data were presented as mean ± standard deviation (*n* ≥ 3). One-way analysis of variance (ANOVA) was performed, followed by Duncan’s multiple comparison tests, to ascertain any statistically significant distinctions (*p* < 0.05) among the various treatments.

## 3. Results

### 3.1. Identification of Metschnikowia Yeast Isolates

A total of 46 target yeast strains of the genus *Metschnikowia* were screened from 417 fresh table grape samples obtained from a wholesale market with different agricultural products in Nanning, Guangxi. All of the screened yeasts had a clear production of pulcherrimin-pigmented halo (Figure S1). Detailed molecular biological analysis revealed that all 46 yeast isolates were identified as *Metschnikowia* strains (Table S1). These were classified into three species: one isolate of *Metschnikowia* sp., one isolate of *M. shanxiensis*, and 44 isolates of *M. pulcherrima*. *M. pulcherrima* was the predominant species, comprising over 95% of the identified isolates.

### 3.2. Antagonistic Activity of Yeast Strains on Culture Media In Vitro

As shown in Table 1 and Figure S2, all the screened isolates of *Metschnikowia* significantly inhibited the growth of *P. digitatum*, *P. italicum* and *G. citri-aurantii* on PDA plates.

To obtain yeasts that have more potent antagonistic effects against the three pathogens, further screening was performed using inhibitory zone widths of ≥11.00 mm for *Metschnikowia* sp. against *P. digitatum* and *P. italicum*, and of ≥16.00 mm for *Metschnikowia* sp. against *G. citri-aurantii*, respectively. The resulting selected *Metschnikowia* yeast strains with excellent in vitro antimicrobial activity include: XX01, XX02, XX04, XX05, XX07, JN01, JN011, JN012, XN05, WM04, WM05, WM10 and WM11. All of these belong to the *Metschnikowia pulcherrima*.

### 3.3. Antagonistic Activity of Yeast Strains on Citrus Fruit In Vivo

**Figure 1:**
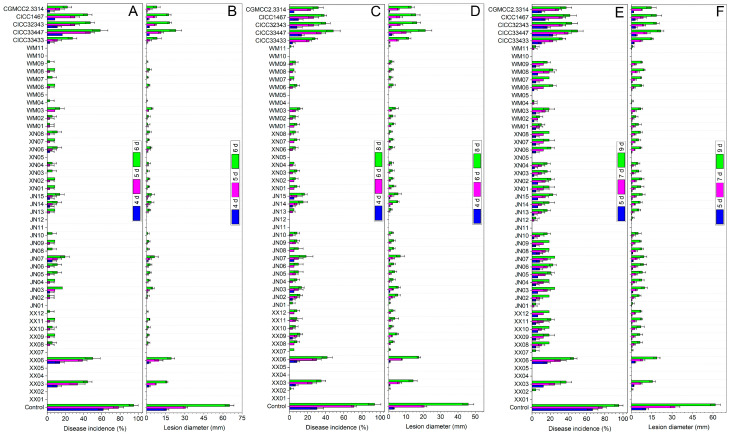
In vivo antagonism of 51 yeast isolates in the control of green mold (**A**,**B**), blue mold (**C**,**D**) and sour rot (**E**,**F**) caused by *P. digitatum*, *P. italicum* and *G. citri-aurantii* on citrus fruit. The columns represent the mean of three replicates and vertical bars show the standard error.

A The DI and LD values (as shown in Figure 1 and Tables S2–S4) suggested that all 51 *Metschnikowia* yeasts tested had a significant inhibitory effect on the green and blue mold, and sour rot, which are caused by *P. digitatum*, *P. italicum*, and *G. citri-aurantii*, respectively, when compared to the control.

In general, among the 46 screened *Metschnikowia* sp. strains, except for the cases of XX03 and XX06, the ability to control the three major diseases in postharvest citrus fruits was superior to that of the five commercially purchased *M. pulcherrima* control strains. During the storage period, all 51 yeast strains, with the exception of XX03 and XX06, decreased the incidence of the three diseases by more than 70%. Among them, XX01, XX04, XX05, JN11, XN05, WM05, and WM10 completely controlled the occurrence of green mold, blue mold, and sour rot throughout the storage period, demonstrating outstanding biocontrol efficacy.

### 3.4. Pulcherrimin Pigment Production of Yeast Isolates

**Figure 2:**
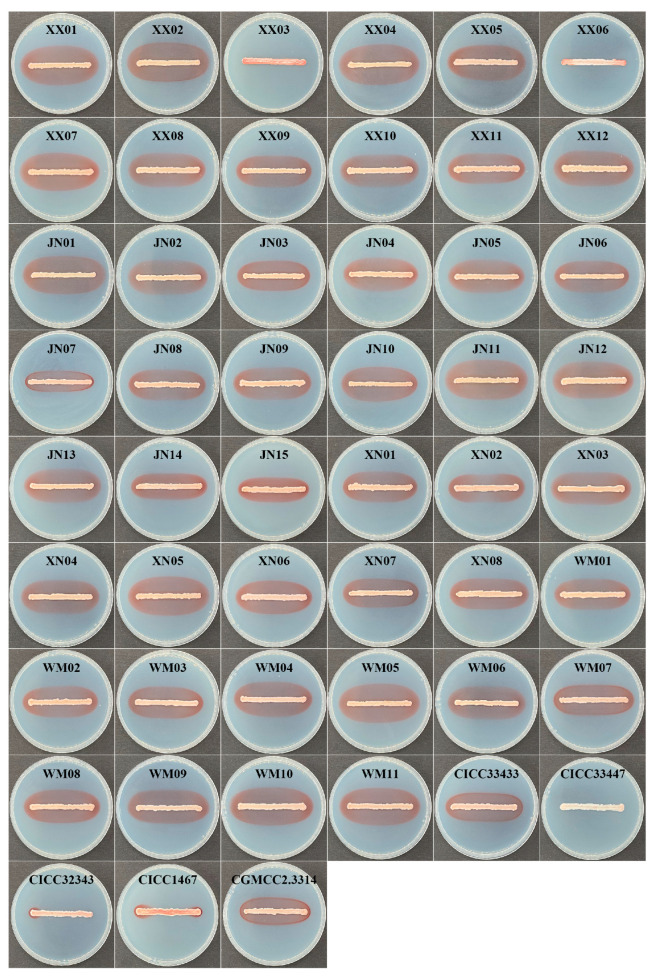
The pulcherrimin pigment produced by 51 *Metschnikowia* yeasts on PDA plate supplemented with 10 mg L^−1^ FeCl_3_.

Except for CICC33447, the colony bands of 51 *Metschnikowia* yeasts exhibited red pigment production on PDA plates containing 10 mg L^−1^ FeCl_3_, while CICC33447 appeared white (Figure 2). Additionally, except for XX03, XX06 and CICC33447, bands of pulcherrimin pigmentation of varying sizes formed around the 48 yeast colonies, with only small pigmented halos produced by CICC32343 and CICC1467.

### 3.5. Iron Competition and Antagonism

For the subsequent experiments, we randomly selected three strains (XX01, XX05, WM05) from a pool of seven *M. pulcherrima* strains (XX01, XX04, XX05, JN11, XN05, WM05, and WM10). These strains have been proven to effectively manage the three major post-harvest citrus diseases during storage, as detailed in Section 3.3. Furthermore, the *M. pulcherrima* s strains XX06 and CICC33447 were also chosen as the control group in the experiment due to their limited and nearly negligible pigment production, respectively (Figure 2).

#### 3.5.1. Subsubsection Effect of Iron on Pigment Production and Antagonism of Yeasts In Vitro

**Figure 3:**
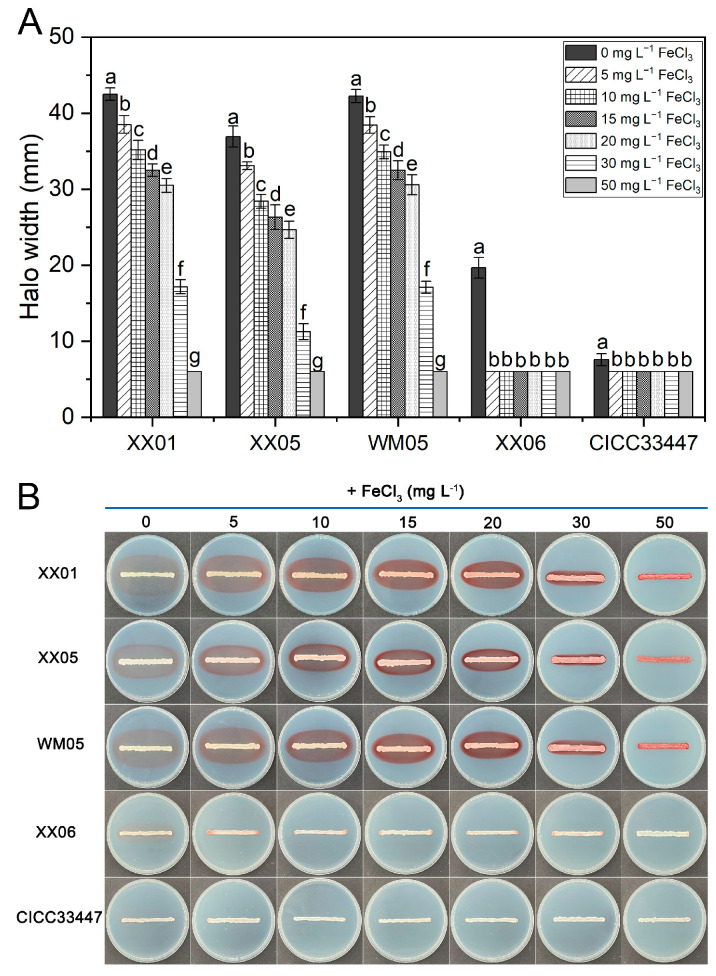
Effect of FeCl_3_ concentration on the width of pulcherrimin-pigmented halos produced by *M. pulcherrima* strains on PDA plates. (**A**) Columns (treatments) represent the mean of three replicates and vertical bars show the standard error of the mean. Different letters indicate statistically significant differences based on Duncan’s multiple range test (*p* < 0.05). (**B**) The photographs show the pigmentation halos of *M. pulcherrima* strains on the FeCl_3_-supplemented PDA plates corresponding to the histogram.

As presented in Figure 3, it could be observed that the increase in FeCl_3_ concentration within the PDA medium resulted in a gradual reduction in the width of the pigment halos produced by the three pulcherrimin-producing *M. pulcherrima* strains. Additionally, the colony band transitioned from white to pink, and ultimately to dark red. At a FeCl_3_ concentration of 50 mg L^−1^, the pigmented halo widths of XX01, XX05, and WM05 coincided with their respective colony band widths. Furthermore, the colony bands exhibited a dark red color, and no pigment ring was observed surrounding the colony in any of the three strains. The *M. pulcherrima* XX06, characterized by its lower intensity of pigment production, produced a narrow-colored halo, and the production of additional pigment bands was halted when the exogenous FeCl_3_ concentration was ≥5 mg L^−1^. For the virtually unpigmented strain CICC33447, no pigment halos were formed under varying concentrations of FeCl_3_.

**Figure 4:**
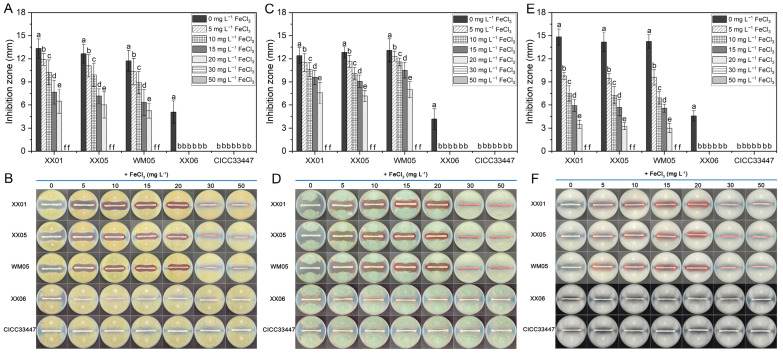
Effect of FeCl_3_ concentration on the width of the inhibition zones by *M. pulcherrima* strains against *P. digitatum* (**A**,**B**), *P. italicum* (**C**,**D**) and *G. citri-aurantii* (**E**,**F**) on PDA plates. Columns (**A**,**C**,**E**) represent the mean of three replicates and vertical bars show the standard error of the mean. Different letters indicate statistically significant differences based on Duncan’s multiple range test (*p* < 0.05). The photographs (**B**,**D**,**F**) show the inhibition areas of *M. pulcherrima* strains on the FeCl_3_-supplemented PDA plates corresponding to the histogram.

Similarly, without the addition of exogenous FeCl_3_, the pulcherrimin-producing *M. pulcherrima* yeasts XX01, XX05, WM05, and XX06 exhibited the widest inhibition zones against *P. digitatum*, *P. italicum*, and *G. citri-aurantii* (Figure 4). However, the size of these inhibition zones noticeably decreased as the concentration of FeCl_3_ increased, and *M. pulcherrima* (XX01, XX05, and WM05) did not exhibit any inhibitory effect on the growth of these three pathogens when the FeCl_3_ concentration was ≥30 mg L^−1^. As for the strain XX06, characterized by its weaker pigment production capability, no inhibitory zones were observed when the FeCl_3_ concentration ≥5 mg L^−1^. CICC33447, which produces almost no pigment, exhibited no substantial inhibitory effects on all three citrus pathogenic fungus, whereas varied doses of FeCl_3_ had no apparent impact on its biocontrol antagonistic efficacy.

#### 3.5.2. Effect of Iron on Biocontrol Efficacy of Yeasts against Postharvest Citrus Pathogens In Vivo

**Figure 5:**
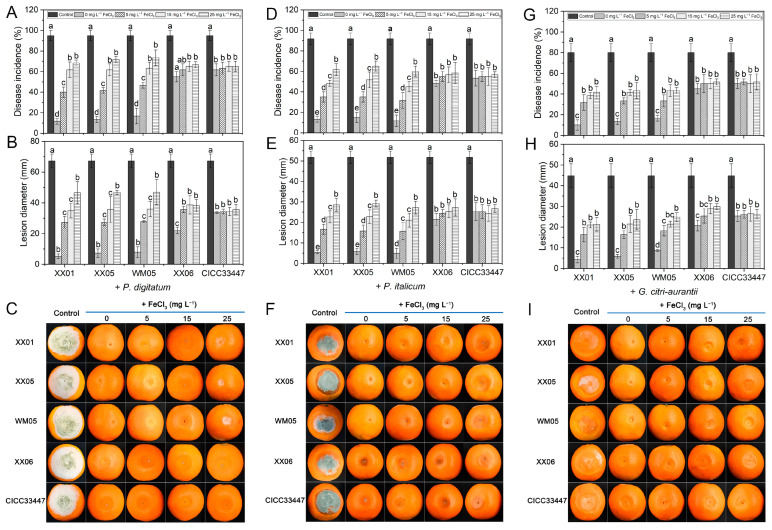
Effect of iron on efficacy of *M. pulcherrima* strains in controlling green mold (**A**–**C**), blue mold (**D**–**F**) and sour rot (**G**–**I**) on citrus fruit. Statistical analysis of disease incidence and lesion diameters are illustrated following the 6 d (infection with *P. digitatum*, **A**,**B**), 8 d (infection with *P. italicum*, **D**,**E**) and 9 d (infection with *G. citri-aurantii*, **G**,**H**) storage, respectively. The pictures showed the biocontrol performance of different treatment groups on the 6th (**C**), 8th (**F**), and 9th (**I**) day after inoculation. The columns represent the mean of three replicates and vertical bars show the standard error. Different letters indicate statistically significant differences based on Duncan’s multiple range test (*p* < 0.05).

Figure 5 shows the effect of iron on the antagonistic activity of *M. pulcherrima* against citrus green mold (A–C), blue mold (D–F), and sour rot (G–I) diseases. The in vivo experiments on citrus fruit showed similar outcomes to those obtained in in vitro studies (Figure 4). The addition of FeCl_3_ to the wounds of fruit treated with three strains of high pigment-producing *M. pulcherrima* resulted in a significant increase in the disease incidence and lesion diameter of green mold, blue mold and sour rot. Within the tested range of 0 to 50 mg L^−1^, the biocontrol activities of the three *M. pulcherrima* strains (XX01, XX05 and WM05) against both citrus green and blue mold significantly decreased as the concentration of FeCl_3_ increased (Figure 5A–F). However, when the FeCl_3_ concentration ranged from 5.0 to 50 mg L^−1^, there was no significant impact on the development of citrus sour rot (Figure 5G–I). This implied that *P. digitatum* and *P. italicum* require a greater iron consumption compared to *G. citri-aurantii* during their growth and infection processes. Different concentrations of FeCl_3_ did not significantly affect the occurrence and development of XX06 and CICC33447 for controlling of citrus green and blue mold, as well as sour rot decay. However, for XX06, the addition of certain concentration of FeCl_3_ (≥5 mg L^−1^) resulted in higher disease incidence and lesion diameter statistics than the control (0 mg L^−1^ of FeCl_3_).

#### 3.5.3. Effect of Iron on the Growth and the PA Production of Selected Yeasts

**Figure 6:**
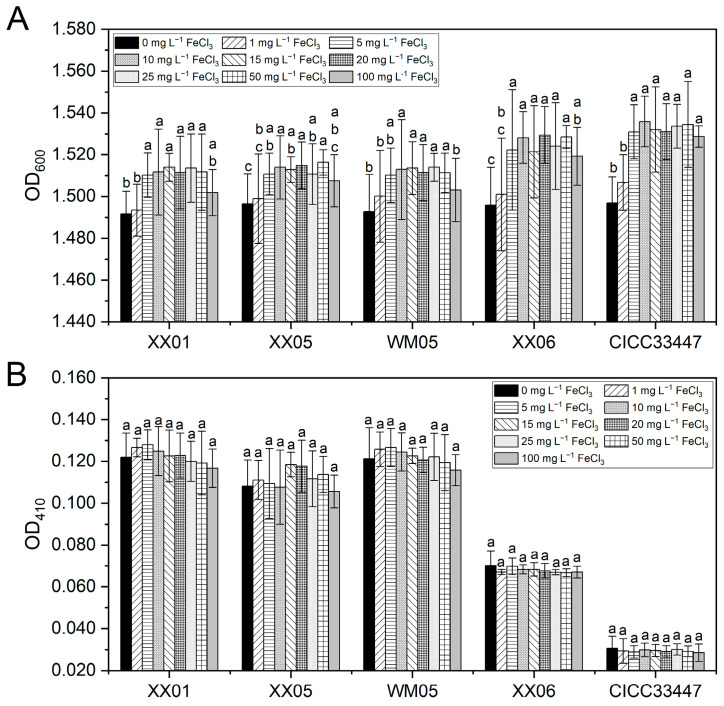
Effect of FeCl_3_ on the cell densities (**A**) and the pulcherriminic acid yield (**B**) of selected *M. pulcherrima* strains. The OD_600_ and OD_410_ of each strain was measured after culture for 30 h. The columns represent the mean of three replicates and vertical bars show the standard error. Different letters indicate statistically significant differences based on Duncan’s multiple range test (*p* < 0.05).

As illustrated in Figure 6A, supplementing the medium with FeCl_3_ enhanced the cell growth of the five *M. pulcherrima* strains. Notably, the addition of 5–50 mg L^−1^ FeCl_3_ markedly stimulated yeast proliferation. PA is a secondary metabolite. FeCl_3_ at the tested concentrations (0–100 mg L^−1^) had no significant effect on PA production by the five yeast strains (Figure 6B).

#### 3.5.4. Sensitivity of *P. digitatum*, *P. italicum* and *G. citri-aurantii* to Iron Starvation

**Figure 7:**
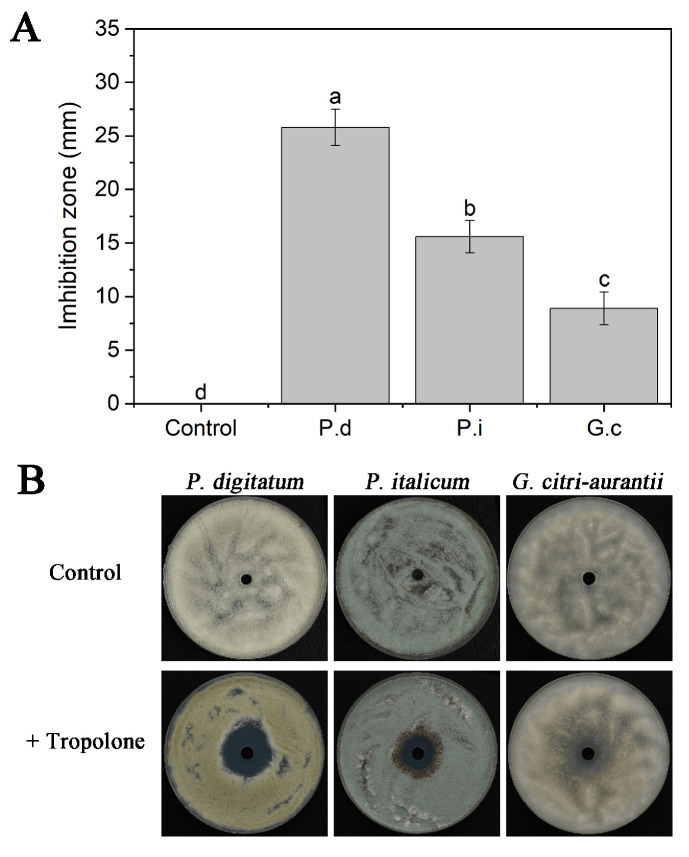
Inhibition of *P. digitatum*, *P. italicum* and *G. citri-aurantii* by tropolone. Columns (**A**) represent the mean of three replicates and vertical bars show the standard error of the mean. Different letters indicate statistically significant differences based on Duncan’s multiple range test (*p* < 0.05). The photograph (**B**) shows the inhibition zones of tropolone activity against *P. digitatum*, *P. italicum* and *G. citri-aurantii*. P.d, *P. digitatum*; P.i, *P. italicum*; G.c, *G. citri-aurantii*.

We selected tropolone, known for its potent affinity for iron ions [11,13,16], for evaluating the susceptibility of *P. digitatum*, *P. italicum*, and *G. citri-aurantii* to iron deprivation. As illustrated in Figure 7, all three pathogens exhibited sensitivity to iron-deficient conditions. Among them, *P. digitatum* exhibited the highest sensitivity, followed by *P. italicum* and *G. citri-aurantii*. These findings aligned closely with the results from the fruit experiment.

## 4. Discussion

In general, PA or pulcherrimin-producing strains can generally serve as ideal candidates for the development of antibacterial and anti-plant-pathogenic fungal agents [20,22]. When tested for pigment production, some *Metschnikowia* species are found to produce characteristic reddish halos known as pulcherrimin and their antagonistic capacity appears well correlated with the size of the red-pigmented zones [17]. In this study, *Metschnikowia* yeasts, originating from the surface of table grapes, were screened in an iron-containing medium based on the characteristics of their colonies that produced the red pulcherrimin pigment. A total of 46 *Metschnikowia* strains were screened and identified. Notably, all 46 strains exhibited distinct pulcherrimin-pigmented halos surrounding their colonies. Among them, *M. pulcherrima* had the largest number of strains, with 44 strains. This was consistent with the previous finding that *M. pulcherrima* strains were frequently found in grapes [5]. On PDA plates containing 10 mg·L^−1^ FeCl_3_, 51 *Metschnikowia* strains were observed to be able to produce pigment circles of different sizes (Figure 2), suggesting that yeasts belonging to the genus *Metschnikowia* display strain-specific differences in pulcherrimin production [18]. All 51 *Metschnikowia* yeasts were highly effective against *P. digitatum*, *P. italicum* and *G. citri-aurantii* in vitro and in vivo on citrus fruits compared with the control (Table 1 and Tables S2–S4, Figure 1 and Figure S2), implying that *Metschnikowia* yeasts with pigment-producing capabilities could potentially be applied in the prevention and control of postharvest diseases of citrus.

A comparison of yeast strains revealed that those with a larger pigment-producing ability (e.g., XX01, XX02, XX04, XX05, XX07, JN01, JN11, JN12, XN05, WM01, WM02, WM04, WM05, WM10, and WM11) showed greater effectiveness in controlling *P. digitatum*, *P. italicum*, and *G. citri-aurantii*. In contrast, yeast strains with less pigment production (e.g., XX03, XX06, and CICC33447) were significantly less antagonistic to these three pathogens (Figure 1 and Figure S2, Tables S2–S4). Closer inspection revealed that there seemed to be a consistent correlation between the boundaries of pigmentation zones and the edges of inhibitory areas. Similar results were also found by Horváth et al. [19].

Iron is a vital element necessary for the growth, proliferation, and pathogenicity of pathogens. It is required as an essential cofactor for numerous enzymes and proteins implicated in pivotal biological processes, such as cellular metabolism and energy generation, etc. [30,31]. Competition for iron has long been recognized as an important biocontrol mechanism employed by antagonists against post-harvest pathogens [32]. Pulcherrimin, an insoluble specific siderophore formed by PA bound Fe^3+^ [33], cannot be recognized and therefore utilized by *P. digitatum*, *P. italicum*, and *G. citri-aurantii*. This leads indirectly to an inhibitory effect on the three pathogens due to a competitive advantage in Fe^3+^ depletion. Upon the introduction of exogenous FeCl_3_ into the PDA culture medium, the pigmentation halos around the yeast colony notably diminished and eventually disappeared with increasing FeCl_3_ concentration. Simultaneously, the inhibitory zone generated against the pathogens also significantly reduced and gradually vanished (Figure 3 and Figure 4). This is due to the fact that as the concentration of Fe^3+^ increases, although the amount of PA produced by the *M. pulcherrima* remains constant (Figure 6), the PA secreted into the extracellular space however cannot diffuse further before it binds with the abundant iron to form stable pulcherrimin near the yeast colonies, leading to progressively narrower bands of pigmentation. Simultaneously, the increasing amount of free Fe^3+^ in the medium was sufficient to meet requirements for the spore germination and mycelial growth of *P. digitatum*, *P. italicum*, and *G. citri-aurantii*. Thus, the inhibitory effect on the pathogens began to decrease gradually until disappearing. For yeast strains that produce weaker pigments (XX06 and CICC33447), lower levels of PA production mean that a lower concentration of iron is sufficient to completely deprive the yeast of its ability to produce the pulcherrimin-pigmented halo and inhibit pathogens (Figure 4 and Figure 5). It is not difficult to perceive that a positive correlation appears to exist between the antagonistic capacity of *M. pulcherrima* and the size of the red pigmented halo. Furthermore, it was noted that even when high concentrations of iron were added to the fruit wounds, the *M. pulcherrima* did not completely lose its effectiveness in controlling green mold, blue mold, and sour rot (Figure 5). This suggested that the biocontrol of citrus diseases by the pulcherrimin-producing *Metschnikowia* yeast involves various mechanisms, in which the competition for iron may play a pivotal role.

In addition, the addition of exogenous Fe^3+^ significantly reduced the effectiveness of *M. pulcherrima* in controlling three postharvest citrus diseases. This is evident from the significant increases in the DI and LD of green mold, blue mold and sour rot with the increase in iron concentration (Figure 5). Moreover, the magnitude of the increase in DI of green and blue mold was higher than that of sour rot, indicating that there are greater requirements for iron in the growth and infection processes of *P. digitatum* and *P. italicum* compared to *G. citri-aurantii*. Oztekin and Karbancioglu-Guler [16] also observed the higher dependency of spore germination and mycelial growth of *P. digitatum* on iron ions compared to *P. expansum*. Different pathogens exhibit varying sensitivities to iron-deficient environments, and our experiments confirmed that *P. digitatum* and *P. italicum* were more sensitive to iron deficiency compared to *G. citri-aurantii* (Figure 7). However, the molecular mechanisms underlying the differences in competition for iron ions between the three pathogens and *M. pulcherrima* need to be further explored.

## 5. Conclusions

In this study, 46 strains of *Metschnikowia* yeasts from the surface of table grapes were screened and identified. The results of antagonism assays showed that all the 46 *Metschnikowia* yeast strains could effectively control the citrus green mold, blue mold and sour rot caused by *P. digitatum*, *P. italicum* and *G. citri-aurantii*, respectively. The pulcherrimin-producing strains of *M. pulcherrima* showed stronger antagonistic properties against the three postharvest citrus pathogens, suggesting that this species has the potential to be utilized as a biocontrol agent. The production of pulcherrimin and the biocontrol efficacy of *M. pulcherrima* strains were directly influenced by the iron concentration, and there appeared to be a positive correlation between antagonistic capacity and the size of the red pigmented halo. By competing for iron ions, pulcherrimin-producing *M. pulcherrima* strains inhibited the growth of *P. digitatum*, *P. italicum* and *G. citri-aurantii* in vitro and in vivo. However, the inhibitory effect may vary depending on the sensitivity of the three pathogens to iron starvation. Additionally, the mechanism of how iron starvation, triggered by iron competition, inhibits the growth of the three pathogens needs to be further investigated.

## Figures and Tables

**Table 1:** In vitro mycelial growth inhibition of 51 yeast isolates of *Metschnikowia* against *P. digitatum*, *P. italicum* and *G. citri-aurantii* on PDA plates.

Isolate	Width of Inhibition Zone (mm)		
	*P. digitatum*	*P. italicum*	*G. citri-aurantii*
XX01	12.60 ± 055	11.00 ± 0.87	16.86 ± 0.69
XX02	12.50 ± 1.22	11.13 ± 0.64	16.75 ± 1.28
XX03	7.00 ± 0.89	5.71 ± 1.25	10.78 ± 0.83
XX04	11.60 ± 1.14	12.43 ± 1.27	16.17 ± 0.75
XX05	12.00 ± 1.00	11.33 ± 0.82	16.13 ± 0.64
XX06	5.43 ± 0.98	3.71 ± 1.38	9.57 ± 1.40
XX07	12.20 ± 1.30	11.29 ± 1.50	17.50 ± 0.55
XX08	10.17 ± 0.75	9.50 ± 1.87	15.57 ± 0.79
XX09	9.00 ± 0.58	8.17 ± 1.94	15.00 ± 0.82
XX10	9.83 ± 0.75	10.14 ± 1.77	15.75 ± 0.89
XX11	9.33 ± 0.52	9.57 ± 1.40	14.00 ± 1.10
XX12	12.40 ± 0.89	10.00 ± 1.53	15.33 ± 0.52
JN01	11.00 ± 0.58	11.17 ± 1.72	16.43 ± 0.53
JN02	10.29 ± 0.76	8.86 ± 1.21	15.14 ± 1.57
JN03	7.83 ± 0.98	8.00 ± 1.12	13.67 ± 1.03
JN04	9.67 ± 0.82	10.00 ± 0.89	15.44 ± 1.88
JN05	9.60 ± 0.55	9.14 ± 1.77	15.22 ± 1.48
JN06	9.20 ± 0.45	9.33 ± 1.37	14.00 ± 0.63
JN07	6.20 ± 0.45	7.88 ± 1.73	13.83 ± 1.47
JN08	10.00 ± 0.71	9.67 ± 0.82	13.67 ± 0.82
JN09	9.83 ± 0.75	9.71 ± 0.76	14.00 ± 1.26
JN10	10.17 ± 0.75	9.83 ± 0.75	15.50 ± 1.05
JN11	11.43 ± 0.79	11.14 ± 1.35	16.43 ± 1.13
JN12	11.14 ± 0.69	11.25 ± 1.49	16.00 ± 0.93
JN13	9.80 ± 1.10	10.38± 1.06	15.50 ± 1.05
JN14	7.60 ± 0.89	8.57 ± 1.40	15.00 ± 0.89
JN15	7.80 ± 1.64	8.71 ± 1.25	14.00 ± 0.63
XN01	8.33 ± 0.52	9.75 ± 1.16	15.14 ± 0.69
XN02	9.00 ± 0.71	10.13 ± 1.13	14.60 ± 0.55
XN03	9.86 ± 0.69	10.00 ± 1.07	15.33 ± 0.52
XN04	9.57 ± 0.53	10.29 ± 0.95	15.43 ± 1.51
XN05	11.13 ± 1.13	11.33 ± 0.82	16.00 ± 0.93
XN06	7.50 ± 1.41	10.14 ± 1.46	14.29 ± 0.76
XN07	7.71 ± 0.76	10.67 ± 0.82	14.67 ± 0.82
XN08	8.40 ± 0.55	10.83 ± 1.83	14.00 ±0.63
WM01	12.00 ± 0.71	9.33 ± 0.98	16.50 ± 0.84
WM02	11.86 ± 0.90	10.00 ± 1.26	17.00 ± 0.89
WM03	10.14 ± 1.21	9.50 ± 1.41	14.86 ± 0.38
WM04	11.00 ± 0.76	11.43 ± 1.51	16.29 ± 1.11
WM05	11.14 ±1.21	12.33 ± 0.52	16.00 ± 1.15
WM06	10.86 ± 1.07	10.00 ± 0.63	14.57 ± 1.27
WM07	10.43 ± 1.27	10.57 ± 0.98	15.43 ± 0.53
WM08	10.17 ± 0.98	10.50 ± 1.38	13.50 ± 1.07
WM09	12.33 ± 0.52	10.33 ± 1.03	14.89 ± 1.36
WM10	13.63 ± 1.19	11.57 ± 0.79	16.57 ± 0.98
WM11	12.43 ± 0.79	11.13 ± 1.25	16.71 ± 0.76
CICC33433	9.40 ± 1.34	9.57 ± 1.62	13.67 ± 1.21
CICC33447	0.86 ± 0.69	0.86 ± 0.69	1.14 ± 0.90
CICC32343	5.75 ± 0.89	6.17 ± 1.17	10.14 ± 1.35
CICC1467	6.83 ± 0.75	7.50 ± 1.22	10.43 ± 0.79
CGMCC2.3314	6.00 ± 1.14	8.33 ± 1.21	11.00 ± 1.20

Each value was presented as the mean ± standard deviation of three replicates.

## Data Availability

Data is contained within the article or Supplementary Material.

## Supplementary Materials

The following supporting information can be downloaded at: https://www.mdpi.com/article/10.3390/foods12234249/s1, Table S1. Identification of the yeast species isolated from the surface of table grape from fruit market in Nanning. Table S2. In vivo antagonism of 51 yeast isolates against green mold caused by *P. digitatum* on citrus fruit. Table S3. In vivo antagonism of 51 yeast isolates against blue mold caused by *P. italicum* on citrus fruit. Table S4. In vivo antagonism of 51 yeast isolates in the control of sour rot caused by *G. citri-aurantii* on citrus fruit. Figure S1: Morphological characteristics of screened *Metschnikowia* yeasts on YPD + Fe medium; Figure S2: Effects of the *Metschnikowia* yeast isolates on the mycelial growth of *P. digitatum*, *P. italicum* and *G. citri-aurantii*.

## References

[1] Chhikara N., Kour R., Jaglan S., Gupta P., Gat Y., Panghal A. (2018). Citrus medica: Nutritional, phytochemical composition and health benefits—A review. Food Funct..

[2] Xu L., Feng L., Sun J., Mao L., Li X., Jiang Y., Duan X., Li T. (2022). Antifungal activities of a natural trisaccharide ester against sour rot in mandarin fruit. Postharvest Biol. Technol..

[3] Zhang X., Li B., Zhang Z., Chen Y., Tian S. (2020). Antagonistic yeasts: A promising alternative to chemical fungicides for controlling postharvest decay of Fruit. J. Fungi.

[4] Wang Z., Sui Y., Li J., Tian X., Wang Q. (2020). Biological control of postharvest fungal decays in citrus: A review. Crit. Rev. Food Sci. Nutr..

[5] Morata A., Loira I., Escott C., del Fresno J.M., Bañuelos M.A., Suárez-Lepe J.A. (2019). Applications of *Metschnikowia pulcherrima* in wine biotechnology. Fermentation.

[6] Kregiel D., Nowacka M., Rygala A., Vadkertiová R. (2022). Biological activity of pulcherrimin from the *Meschnikowia pulcherrima* Clade. Molecules.

[7] Hilber-Bodmer M., Schmid M., Ahrens C.H., Freimoser F.M. (2017). Competition assays and physiological experiments of soil and phyllosphere yeasts identify *Candida subhashii* as a novel antagonist of filamentous fungi. BMC Microbiol..

[8] Leverentz B., Conway W.S., Janisiewicz W., Abadias M., Kurtzman C.P., Camp M.J. (2006). Biocontrol of the food-borne pathogens *Listeria monocytogenes* and *Salmonella enterica* serovar Poona on fresh-cut apples with naturally occurring bacterial and yeast antagonists. Appl. Environ. Microbiol..

[9] Fadahunsi I.F., Olubodun S. (2021). Antagonistic pattern of yeast species against some selected food-borne pathogens. Bull. Natl. Res. Cent..

[10] Tian Y.Q., Li W., Jiang Z.T., Jing M.M., Shao Y.Z. (2018). The preservation effect of *Metschnikowia pulcherrima* yeast on anthracnose of postharvest mango fruits and the possible mechanism. Food Sci. Biotechnol..

[11] Saravanakumar D., Ciavorella A., Spadaro D., Garibaldi A., Gullino M.L. (2008). *Metschnikowia pulcherrima* strain MACH1 outcompetes *Botrytis cinerea*, *Alternaria alternata* and *Penicillium expansum* in apples through iron depletion. Postharvest Biol. Technol..

[12] Yang H., Wang L., Li S., Gao X., Wu N., Zhao Y., Sun W. (2021). Control of postharvest grey spot rot of loquat fruit with *Metschnikowia pulcherrima* E1 and potential mechanisms of action. Biol. Control.

[13] Sipiczki M. (2006). *Metschnikowia* strains isolated from botrytized grapes antagonize fungal and bacterial growth by iron depletion. Appl. Environ. Microbiol..

[14] Pawlikowska E., James S.A., Breierova E., Antolak H., Kregiel D. (2019). Biocontrol capability of local *Metschnikowia* sp. isolates. Antonie Van. Leeuwenhoek.

[15] Parafati L., Vitale A., Restuccia C., Cirvilleri G. (2015). Biocontrol ability and action mechanism of food-isolated yeast strains against *Botrytis cinerea* causing post-harvest bunch rot of table grape. Food Microbiol..

[16] Oztekin S., Karbancioglu-Guler F. (2021). Bioprospection of *Metschnikowia* sp. isolates as biocontrol agents against postharvest fungal decays on lemons with their potential modes of action. Postharvest Biol. Technol..

[17] Sipiczki M. (2020). *Metschnikowia pulcherrima* and related pulcherrimin-producing yeasts: Fuzzy species boundaries and complex antimicrobial antagonism. Microorganisms.

[18] Pawlikowska E., Kolesińska B., Nowacka M., Kregiel D. (2020). A new approach to producing high yields of pulcherrimin from *Metschnikowia* yeasts. Fermentation.

[19] Horváth E., Dályai L., Szabó E., Barna T., Kalmár L., Posta J., Sipiczki M., Csoma H., Miklós I. (2021). The antagonistic *Metschnikowia andauensis* produces extracellular enzymes and pulcherrimin, whose production can be promoted by the culture factors. Sci. Rep..

[20] Yuan S., Yong X., Zhao T., Li Y., Liu J. (2020). Research progress of the biosynthesis of natural bio-antibacterial agent pulcherriminic acid in *Bacillus*. Molecules.

[21] Kregiel D., Czarnecka-Chrebelska K.H., Schusterová H., Vadkertiová R., Nowak A. (2023). The *Metschnikowia pulcherrima* clade as a model for assessing inhibition of *Candida* spp. and thetoxicity of its metabolite, pulcherrimin. Molecules.

[22] Wang S., Wang H., Zhang D., Li X., Zhu J., Zhan Y., Cai D., Wang Q., Ma X., Wang D. (2020). Multistep metabolic engineering of *Bacillus licheniformis* to improve pulcherriminic acid production. Appl. Environ. Microbiol..

[23] Öztekin S., Karbancioglu-Guler F. (2023). Biological control of green mould on mandarin fruit through the combined use of antagonistic yeasts. Biol. Control.

[24] Rodriguez Assaf L.A., Pedrozo L.P., Nally M.C., Pesce V.M., Toro M.E., Castellanos de Figueroa L.I., Vazquez F. (2020). Use of yeasts from different environments for the control of *Penicillium expansum* on table grapes at storage temperature. Int. J. Food Microbiol..

[25] Wang S., Zhang H., Ruan C., Yi L., Deng L., Zeng K. (2021). *Metschnikowia citriensis* FL01 antagonize *Geotrichum citri-aurantii* in citrus fruit through key action of iron depletion. Int. J. Food Microbiol..

[26] Liu Y., Yao S., Deng L., Ming J., Zeng K. (2019). Different mechanisms of action of isolated epiphytic yeasts against *Penicillium digitatum* and *Penicillium italicum* on citrus fruit. Postharvest Biol. Technol..

[27] Wang S., Ruan C., Yi L., Deng L., Yao S., Zeng K. (2020). Biocontrol ability and action mechanism of *Metschnikowia citriensis* against *Geotrichum citri-aurantii* causing sour rot of postharvest citrus fruit. Food Microbiol..

[28] Wang D., Zhan Y., Cai D., Li X., Wang Q., Chen S. (2018). Regulation of the synthesis and secretion of the iron chelator cyclodipeptide pulcherriminicacid in *Bacillus licheniformis*. Appl. Environ. Microbiol..

[29] Wang S., Zhang H., Qi T., Deng L., Yi L., Zeng K. (2022). Influence of arginine on the biocontrol efficiency of *Metschnikowia citriensis* against *Geotrichum citri-aurantii* causing sour rot of postharvest citrus fruit. Food Microbiol..

[30] Zhang J., Liu B., Li M., Feng D., Jin H., Wang P., Liu J., Xiong F., Wang J., Wang H.-B. (2015). The bHLH transcription factor bHLH104 Interacts with IAA-LEUCINE RESISTANT3 and modulates iron homeostasis in Arabidopsis. Plant Cell.

[31] Qin Y., He Y., She Q., Larese-Casanova P., Li P., Chai Y. (2019). Heterogeneity in respiratory electron transfer and adaptive iron utilization in a bacterial biofilm. Nat. Commun..

[32] Dukare A.S., Paul S., Nambi V.E., Gupta R.K., Singh R., Sharma K., Vishwakarma R.K. (2019). Exploitation of microbial antagonists for the control of postharvest diseases of fruits: A review. Crit. Rev. Food Sci. Nutr..

[33] Krause D.J., Kominek J., Opulente D.A., Shen X.X., Zhou X., Langdon Q.K., DeVirgilio J., Hulfachor A.B., Kurtzman C.P., Rokas A. (2018). Functional and evolutionary characterization of a secondary metabolite gene cluster in budding yeasts. Proc. Natl. Acad. Sci. USA.

