# Supplementary material for: Iron Competition as an Important Mechanism of Pulcherrimin-Producing Metschnikowia sp. Strains for Controlling Postharvest Fungal Decays on Citrus Fruit
Source: Foods. 2023 Nov 24;12(23):4249. doi: 10.3390/foods12234249 (PMC10706177; doi:10.3390/foods12234249)
Supplement: Supplementary file 1 [file foods-12-04249-s001.zip › foods-2651963-supplementary.pdf]

Table S1. Identification of the yeast species isolated from the surface of table grape from fruit market in Nanning.

Isolate ID	[GenBank accession number]	Identity (%)
XX01	*Metschnikowia pulcherrima* isolate 3Y138 [MT225539.1]	99.26
XX02	*Metschnikowia* cf. *pulcherrima* isolate 7H82P1-7 [MT749270.1]	99.26
XX03	*Metschnikowia* cf. *pulcherrima* isolate 4H82P2-1 [MT749265.1]	99.26
XX04	*Metschnikowia pulcherrima* culture CBS:5833 [KY108497.1]	99.45
XX05	*Metschnikowia pulcherrima* culture CBS:2253 [KY108494.1]	99.63
XX06	*Metschnikowia* cf. *pulcherrima* isolate 4H82P2-1 [MT749265.1]	99.08
XX07	*Metschnikowia pulcherrima* culture CBS:5833 [KY108497.1]	99.26
XX08	*Metschnikowia pulcherrima* culture CBS:2243 [KY108490.1]	99.45
XX09	*Metschnikowia* cf. *pulcherrima* isolate 4H82P2-1 [MT749265.1]	99.45
XX10	*Metschnikowia pulcherrima* culture CBS:2243 [KY108490.1]	99.45
XX11	*Metschnikowia* sp. 11-1089 clone 22a[KM275372.1]	99.08
XX12	*Metschnikowia pulcherrima* culture CBS:2243 [KY108490.1]	99.45
JN01	*Metschnikowia pulcherrima* culture CBS:5833[KY108497.1]	99.63
JN02	*Metschnikowia pulcherrima* culture CBS:2243 [KY108490.1]	99.45
JN03	*Metschnikowia aff. pulcherrima* P01A016 [JX188181.1]	99.82
JN04	*Metschnikowia pulcherrima culture* CBS:2243 [KY108490.1]	99.45
JN05	*Metschnikowia pulcherrima culture* CBS:2243 [KY108490.1]	99.45
JN06	*Metschnikowia* aff. *pulcherrima* P01A016 [JX188181.1]	99.63
JN07	*Metschnikowia* cf. pulcherrima isolate 4H82P2-1 [MT749265.1]	99.26
JN08	*Metschnikowia pulcherrima* culture CBS:610 [KY108493.1]	99.63
JN09	*Metschnikowia* cf. *pulcherrima* isolate 2H82P3-1 [MT749261.1]	99.45
JN10	*Metschnikowia* cf. *pulcherrima* isolate 4H82P2-1 [MT749265.1]	99.45
JN11	*Metschnikowia pulcherrima* culture CBS:5833 [KY108497.1]	99.63
JN12	*Metschnikowia pulcherrima* culture CBS:2243 [KY108490.1]	99.45
JN13	*Metschnikowia pulcherrima* culture CBS:2243 [KY108490.1]	99.45
JN14	*Metschnikowia shanxiensis* CBS 10359 [NG_058344.1]	98.52
JN15	*Metschnikowia* cf. *pulcherrima* isolate 7H82P1-7 [MT749270.1]	99.26
XN01	*Metschnikowia pulcherrima* culture CBS:2256 [KY108498.1]	99.63
XN02	*Metschnikowia* aff. *pulcherrima* strain APC 1.2 [CP034458.1]	99.45
XN03	*Metschnikowia pulcherrima* culture CBS:2243 [KY108490.1]	99.45
XN04	*Metschnikowia pulcherrima* culture CBS:2243 [KY108490.1]	99.45
XN05	*Metschnikowia pulcherrima* culture CBS:5833 [KY108497.1]	99.63
XN06	*Metschnikowia pulcherrima* culture CBS:5833 [KY108497.1]	99.63
XN07	*Metschnikowia pulcherrima* isolate 3Y138B [MT225539.1]	99.45
XN08	*Metschnikowia pulcherrima* strain CEC RCF-0-39 [JX103182.1]	99.26
WM01	*Metschnikowia pulcherrima* strain NRRL Y-7111 [JX188179.1]	99.82
WM02	*Metschnikowia* aff. *pulcherrima* P01A016 [JX188181.1]	99.63
WM03	*Metschnikowia pulcherrima* strain NRRL Y-7111 [JX188179.1]	99.63
WM04	*Metschnikowia* aff. *pulcherrima* P01A016 [JX188181.1]	99.63
WM05	*Metschnikowia pulcherrima* culture CBS:5833 [KY108497.1]	99.63
WM06	*Metschnikowia pulcherrima* culture CBS:2243 [KY108490.1]	99.45
WM07	*Metschnikowia* cf. *pulcherrima* isolate 7H82P1-7 [MT749270.1]	99.45
WM08	*Metschnikowia* cf. *pulcherrima* isolate 7H82P1-7 [MT749270.1]	99.45
WM09	*Metschnikowia pulcherrima* culture CBS:610 [KY108493.1]	99.82
WM10	*Metschnikowia* cf. *pulcherrima* isolate 4H82P2-1 [MT749265.1]	99.45
WM11	*Metschnikowia* cf. *pulcherrima* isolate 4H82P2-1 [MT749265.1]	99.45

Table S2. *In vivo* antagonism of 51 yeast isolates against green mold caused by *P. digitatum* on citrus fruit.

Isolate	DI (%)			LD (mm)		
	4 d	5 d	6 d	4 d	5 d	6 d
Control	61.67 ± 5.77	78.33 ± 5.77	95.00 ± 5.00	15.67 ± 1.98	30.69 ± 1.49	65.15 ± 3.44
XX01	0	0	0	0	0	0
XX02	0	0	0	0	0	0
XX03	11.11 ± 4.81	33.33 ± 8.33	44.44 ± 4.81	2.06 ± 0.84	7.26 ± 0.30	15.96 ± 0.84
XX04	0	0	0	0	0	0
XX05	0	0	0	0	0	0
XX06	13.89 ± 4.81	38.89 ± 4.81	50.00 ± 8.33	2.17 ± 1.28	9.78 ± 3.19	19.40 ± 2.76
XX07	0	0	0	0	0	0
XX08	0	2.78 ± 4.81	5.56 ± 4.81	0	0.40 ± 0.70	1.33 ± 1.35
XX09	0	2.78 ± 4.81	8.33 ± 0.00	0	0.26 ± 0.46	1.68 ± 0.84
XX10	0	2.78 ± 4.81	5.56 ± 4.81	0	0.32 ± 0.55	1.50 ± 1.50
XX11	0	2.78 ± 4.81	8.33 ± 0.00	0	0.35 ± 0.60	2.42 ± 0.26
XX12	0	0	2.78 ± 4.81	0	0	0.50 ± 0.87
JN01	0	0	0	0	0	0
JN02	0	2.78 ± 4.81	2.78 ± 4.81	0	0.28 ± 0.48	0.89 ± 1.54
JN03	2.78 ± 4.81	8.33 ± 0.00	16.67 ± 0.00	0.38 ± 0.65	1.65 ± 0.92	4.86 ± 1.25
JN04	0	2.78 ± 4.81	8.33 ± 0.00	0	0.46 ± 0.79	2.28 ±0.68
JN05	0	2.78 ± 4.81	11.11 ± 4.81	0	0.64 ± 1.11	2.82 ± 1.01
JN06	0	2.78 ± 4.81	11.11 ± 4.81	0	0.53 ± 0.91	2.89 ± 1.33
JN07	5.56 ± 4.81	11.11 ± 4.81	19.44 ± 4.81	0.86 ± 0.76	2.68 ± 1.28	6.26 ± 2.88
JN08	0	0	5.56 ± 4.81	0	0	1.35 ± 1.23
JN09	0	2.78 ± 4.81	8.33 ± 0.00	0	0.35 ± 0.60	1.92 ± 0.93
JN10	0	0	5.56 ± 4.81	0	0	1.07 ± 1.06
JN11	0	0	0	0	0	0
JN12	0	0	0	0	0	0
JN13	0	2.78 ± 4.81	8.33 ± 0.00	0	0.58 ± 1.01	2.40 ± 0.99

JN14	2.78 ± 4.81	5.56 ± 4.81	11.11 ± 4.81	0.42 ± 0.72	1.44 ± 1.36	3.63 ± 2.28
JN15	2.78 ± 4.81	5.56 ± 4.81	13.89 ± 4.81	0.36 ± 0.63	1.53 ± 1.38	3.94 ± 2.40
XN01	0	2.78 ± 4.81	8.33 ± 0.00	0	0.26 ± 0.46	1.92 ± 0.61
XN02	0	2.78 ± 4.81	8.33 ± 0.00	0	0.38 ± 0.65	2.10 ± 0.68
XN03	0	0	5.56 ± 4.81	0	0	1.24 ± 1.10
XN04	0	2.78 ± 4.81	5.56 ± 4.81	0	0.63 ± 1.08	1.56 ± 1.65
XN05	0	0	0	0	0	0
XN06	2.78 ± 4.81	5.56 ± 4.81	11.11 ± 4.81	0.43 ± 0.75	1.40 ± 1.64	3.47 ± 0.80
XN07	0	2.78 ± 4.81	8.33 ± 0.00	0	0.24 ± 0.41	1.86 ± 0.61
XN08	0	2.78 ± 4.81	11.11 ± 4.81	0	0.46 ± 0.79	2.39 ± 1.35
WM01	0	2.78 ± 4.81	2.78 ± 4.81	0	0.53 ± 0.91	1.01 ± 1.76
WM02	0	2.78 ± 4.81	5.56 ± 4.81	0	0.33 ± 0.58	1.56 ± 1.40
WM03	0	8.33 ± 0.00	13.89 ± 4.81	0	1.43 ± 0.25	4.42 ± 0.73
WM04	0	0	2.78 ± 4.81	0	0	0.35 ± 0.60
WM05	0	0	0	0	0	0
WM06	0	2.78 ± 4.81	8.33 ± 0.00	0	0.44 ± 0.77	2.19 ± 0.55
WM07	0	0	5.56 ± 4.81	0	0	0.97 ±0.87
WM08	0	2.78 ± 4.81	8.33 ± 0.00	0	0.63 ± 1.08	2.58 ± 1.33
WM09	0	0	2.78 ± 4.81	0	0	0.44 ± 0.77
WM10	0	0	0	0	0	0
WM11	0	0	0	0	0	0
CICC33433	2.78 ± 4.81	11.11 ± 4.81	27.78 ± 4.81	0.39 ± 0.67	2.49 ± 1.65	8.36 ± 3.24
CICC33447	16.67 ± 0.00	47.22 ± 4.81	58.33± 8.33	2.24 ± 0.02	11.40 ± 1.96	23.01 ± 4.57
CICC32343	11.11 ± 4.81	30.56 ± 4.81	47.22 ± 4.81	2.01 ± 0.42	7.08 ± 0.99	18.17 ± 1.45
CICC1467	8.33 ± 0.00	30.56 ± 4.81	44.44 ± 4.81	1.47 ± 0.65	6.72 ± 1.43	17.44 ± 1.80
CGMCC2.3314	2.78 ± 4.81	13.89 ± 4.81	22.22 ± 4.81	0.31 ± 0.53	2.76 ± 1.63	8.15 ± 2.53

* DI% and LD (mm) is shown after 4 d, 5d and 6d (25 °C) of incubation. Each value is presented as the mean ± standard deviation.

Table S3. *In vivo* antagonism of 51 yeast isolates against blue mold caused by *P. italicum* on citrus fruit.

Isolate	DI (%)			LD (mm)		
	4 d	6 d	8 d	4 d	6 d	8 d
Control	30.30 ± 6.94	71.21 ± 2.62	93.94 ± 6.94	2.99 ± 0.62	20.65 ± 1.41	46.02 ± 3.18
XX01	0	0	0	0	0	0
XX02	0	0	1.67 ± 2.89	0	0	0.36 ± 0.62
XX03	6.67 ± 2.89	25.00 ± 5.00	35.00 ± 5.00	0.52 ± 0.38	6.32 ± 1.34	14.13 ± 2.41
XX04	0	0	0	0	0	0
XX05	0	0	0	0	0	0
XX06	8.33 ± 2.89	30.00 ± 5.00	41.67 ± 5.77	0.79 ± 0.25	7.64 ± 0.40	17.46 ± 1.02
XX07	0	0	5.00 ± 0.00	0	0	0.81 ± 0.34
XX08	0	5.00 ± 0.00	8.33 ± 2.89	0	0.81 ± 0.20	2.67 ± 0.70
XX09	3.33 ± 2.89	8.33 ± 2.89	11.67 ± 2.89	0.23 ± 0.23	1.97 ± 0.29	4.89 ± 0.95
XX10	0	3.33 ± 2.89	6.67 ± 2.89	0	0.68 ± 0.64	2.14 ± 0.85
XX11	0	6.67 ± 2.89	8.33 ± 5.77	0	1.45 ± 0.85	3.59 ± 2.26
XX12	0	5.00 ± 0.00	8.33 ± 2.89	0	0.95 ± 0.30	2.70 ± 0.95
JN01	0	0	3.33 ± 2.89	0	0	0.59 ± 0.64
JN02	3.33 ± 2.89	8.33 ± 2.89	11.67 ± 2.89	0.49 ± 0.48	2.58 ± 1.02	5.35 ± 1.41
JN03	5.00 ± 0.00	10.00 ± 5.00	13.33 ± 2.89	0.68 ± 0.09	2.41 ± 0.47	5.57 ± 1.27
JN04	0	3.33 ± 2.89	8.33 ± 2.89	0	0.78 ± 0.78	2.64 ± 1.33
JN05	0	6.67 ± 2.89	10.00 ± 5.00	0	1.17 ± 0.46	3.49 ± 1.27
JN06	0	5.00 ± 0.00	10.00 ± 5.00	0	0.94 ± 0.39	2.88 ± 1.13
JN07	5.00 ± 0.00	10.00 ± 5.00	18.33 ± 7.64	0.58 ± 0.15	2.69 ± 0.87	6.79 ± 2.37
JN08	0	5.00 ± 0.00	10.00 ± 5.00	0	0.87 ± 0.12	2.96 ± 0.84
JN09	0	6.67 ± 2.89	8.33 ± 2.89	0	1.05 ± 0.37	2.88 ± 1.00
JN10	0	3.33 ± 2.89	8.33 ± 2.89	0	0.49 ± 0.44	2.26 ± 0.63
JN11	0	0	0	0	0	0
JN12	0	0	0	0	0	0
JN13	0	3.33 ± 2.89	5.00 ± 0.00	0	0.43 ± 0.38	1.50 ± 0.69
JN14	3.33 ± 2.89	8.33 ± 2.89	15.00 ± 5.00	0.40 ± 0.41	2.08 ± 0.22	5.33 ± 0.71

JN15	5.00 ± 0.00	8.33 ± 5.77	16.67 ± 2.89	0.57 ± 0.25	2.03 ± 1.01	5.48 ± 1.90
XN01	0	5.00 ± 0.00	8.33 ± 2.89	0	1.07 ± 0.34	3.18 ± 1.04
XN02	0	5.00 ± 0.00	6.67 ± 2.89	0	0.85 ± 0.34	2.35 ± 0.74
XN03	0	3.33 ± 2.89	8.33 ± 2.89	0	0.43 ± 0.41	2.27 ± 1.02
XN04	0	3.33 ± 2.89	5.00 ± 0.00	0	0.78 ± 0.78	1.79 ± 1.10
XN05	0	0	0	0	0	0
XN06	0	3.33 ± 2.89	6.67 ± 2.89	0	0.66 ± 0.69	2.27 ± 1.33
XN07	0	5.00 ± 0.00	8.33 ± 2.89	0	0.75 ± 0.40	2.39 ± 0.68
XN08	0	1.67 ± 2.89	6.67 ± 2.89	0	0.23 ± 0.40	1.48 ± 0.92
WM01	0	5.00 ± 0.00	8.33 ± 2.89	0	1.07 ± 0.30	2.96 ± 0.85
WM02	0	3.33 ± 2.89	6.67 ± 2.89	0	0.60 ± 0.66	2.38 ± 1.36
WM03	0	6.67 ± 2.89	11.67 ± 2.89	0	1.42 ± 0.46	3.98 ± 1.61
WM04	0	0	0	0	0	0
WM05	0	0	0	0	0	0
WM06	0	3.33 ± 2.89	8.33 ± 2.89	0	0.87 ± 0.75	2.69 ± 1.32
WM07	0	1.67 ± 2.89	5.00 ± 0.00	0	0.42 ± 0.72	1.58 ± 0.93
WM08	0	3.33 ± 2.89	5.00 ± 0.00	0	0.75 ± 0.65	2.13 ± 0.74
WM09	0	3.33 ± 2.89	6.67 ± 2.89	0	0.66 ± 0.73	2.02 ± 1.03
WM10	0	0	0	0	0	0
WM11	0	0	1.67 ± 2.89	0	0	0.32 ± 0.55
CICC33433	3.33 ± 2.89	21.67 ± 2.89	28.33 ± 2.89	0.41 ± 0.36	5.26 ± 0.66	11.63 ± 1.32
CICC33447	13.33 ± 2.89	35.00 ± 5.00	48.33 ± 7.64	1.91 ± 0.72	10.22± 2.65	21.16 ± 3.83
CICC32343	8.33 ± 5.77	26.67 ± 5.77	40.00 ± 5.00	0.95 ± 0.53	6.78 ± 1.48	16.23 ± 2.26
CICC1467	10.00 ± 5.00	26.67 ± 5.77	38.33 ± 2.89	0.97 ± 0.49	6.49 ± 1.72	15.66 ± 2.48
CGMCC2.3314	5.00 ± 0.00	23.33± 2.89	31.67 ± 5.77	0.56 ± 0.13	5.58 ± 0.54	13.12 ± 1.70

* DI% and LD (mm) is shown after 4 d, 6 d and 8 d (25 °C) of incubation. Each value is presented as the mean ± standard deviation.

Table S4. *In vivo* antagonism of 51 yeast isolates in the control of sour rot caused by *G. citri-aurantii* on citrus fruit.

**Isolate**	**DI (%)**			**LD (mm)**		
	5 d	7 d	9 d	5 d	7 d	9 d
Control	66.67 ± 5.77	77.27 ± 4.55	95.45 ± 4.55	10.69 ± 2.13	32.26 ± 3.48	61.74 ± 3.73
XX01	0	0	0	0	0	0
XX02	0	0	4.17 ± 3.61	0	0	0.64 ± 0.69
XX03	12.50 ± 0.00	25.00 ± 6.25	37.50 ± 6.25	1.79 ± 0.31	7.20 ± 0.80	15.72 ± 2.08
XX04	0	0	0	0	0	0
XX05	0	0	0	0	0	
XX06	16.67 ± 3.61	31.25 ± 6.25	45.83 ± 3.61	2.77 ± 0.62	8.50 ± 1.57	18.80 ± 2.33
XX07	0	0	4.17 ± 3.61	0	0	0.64 ± 0.69
XX08	4.17 ± 3.61	10.42 ± 3.61	18.75 ± 0.00	0.42 ± 0.39	2.40 ± 0.81	6.49 ±1.81
XX09	4.17 ± 3.61	12.50 ± 0.00	18.75 ± 6.25	0.34 ± 0.32	2.32 ± 0.66	6.38 ± 2.03
XX10	6.25 ± 0.00	10.42 ± 3.61	18.75 ± 0.00	0.42 ± 0.05	2.65 ± 1.41	6.92 ± 2.67
XX11	4.17 ± 3.61	12.50 ± 6.25	20.83 ± 3.61	0.35 ± 0.36	2.98 ± 0.99	7.59 ± 0.91
XX12	6.25 ± 0.00	12.50 ± 0.00	18.75 ± 0.00	0.51 ± 0.19	2.79 ± 0.56	7.10 ±0.78
JN01	0	2.08 ± 3.61	4.17 ± 3.61	0	0.14 ± 0.23	1.04 ± 1.22
JN02	4.17 ± 3.61	8.33 ± 3.61	18.75 ± 0.00	0.34 ± 0.30	2.04 ± 0.82	5.90 ± 1.25
JN03	6.25 ± 0.00	16.67 ± 3.61	25.00 ± 0.00	0.92 ± 0.34	3.86 ± 0.86	9.80 ± 2.08
JN04	4.17 ± 3.61	12.50 ± 6.25	18.75 ± 0.00	0.44 ± 0.38	2.65 ± 1.18	7.71 ± 1.72
JN05	6.25 ± 0.00	14.58 ± 7.22	20.83 ± 3.61	0.58 ± 0.15	3.52 ± 0.99	8.21 ± 2.44
JN06	8.33 ± 3.61	16.67 ± 3.61	22.92 ± 3.61	0.77 ± 0.42	3.64 ±1.26	8.97 ± 2.34
JN07	8.33 ± 3.61	16.67 ± 3.61	25.00 ± 0.00	1.21 ± 0.24	4.53 ± 1.33	9.26 ± 2.20
JN08	8.33 ± 3.61	14.58 ± 3.61	18.75 ± 0.00	0.88 ± 0.33	3.38 ± 1.36	7.77 ± 1.27
JN09	4.17 ± 3.61	12.50 ± 0.00	18.75 ± 0.00	0.41 ± 0.38	2.40 ± 0.44	6.79 ± 0.74
JN10	2.08 ± 3.61	8.33 ± 4.81	16.67 ± 3.61	0.24 ± 0.41	1.64 ± 1.39	5.11 ± 2.22
JN11	0	0	0	0	0	0
JN12	0	2.08 ± 3.61	4.17 ± 3.61	0	0.16 ± 0.27	0.99 ± 1.11
JN13	2.08 ± 3.61	10.42 ± 3.61	16.67 ± 3.61	0.16 ± 0.27	1.47 ± 0.72	5.64 ± 1.34
JN14	6.25 ± 0.00	10.42 ± 3.61	18.75 ± 6.25	0.51 ± 0.10	2.64 ± 0.55	6.59 ± 1.44

JN15	6.25 ± 0.00	14.58 ± 3.61	22.92 ± 3.61	0.63 ± 0.14	3.21 ± 0.75	8.23 ± 1.96
XN01	4.17 ± 3.61	12.50 ± 6.25	18.75 ± 6.25	0.40 ± 0.35	2.39 ± 0.81	6.98 ± 2.39
XN02	6.25 ± 0.00	14.58 ± 3.61	20.83 ± 3.61	0.60 ± 0.12	3.18 ± 0.83	7.77 ± 1.63
XN03	2.08 ± 3.61	10.42 ± 3.61	16.67± 3.61	0.16 ± 0.27	1.81 ± 1.08	5.53 ± 1.63
XN04	2.78 ± 3.61	10.42 ± 3.61	16.61± 3.61	0.17 ± 0.29	1.76 ± 1.30	5.01 ± 1.31
XN05	0	0	0	0	0	0
XN06	4.17 ± 3.61	12.50 ± 0.00	20.83 ± 3.61	0.35 ±0.31	2.46 ± 0.30	6.97 ± 0.87
XN07	4.17 ± 3.61	12.50 ± 0.00	18.75 ± 6.25	0.41 ± 0.37	2.44 ± 0.39	5.41 ± 1.71
XN08	4.17 ± 3.61	12.50 ± 0.00	18.75 ± 0.00	0.41 ± 0.35	2.78 ± 0.69	6.83 ± 1.32
WM01	4.17 ± 3.61	8.33 ± 3.61	10.42 ± 3.61	0.36 ± 0.36	2.36 ± 0.97	5.35 ± 2.07
WM02	2.08 ± 3.61	6.25 ± 0.00	8.33 ± 3.61	0.24 ± 0.41	1.32 ± 0.74	3.44 ± 1.55
WM03	4.17 ± 3.61	14.58 ± 3.61	18.75 ± 6.25	0.41 ± 0.37	2.84 ± 0.89	6.93 ± 1.44
WM04	0	2.08 ± 3.61	2.08 ± 3.61	0	0.15 ± 0.25	0.65 ± 1.12
WM05	0	0	0	0	0	0
WM06	2.08 ± 3.61	12.50 ± 0.00	22.92 ± 3.61	0.27 ± 0.47	2.26 ± 0.82	7.69 ± 1.19
WM07	4.17 ± 3.61	12.50 ± 0.00	18.75 ± 0.00	0.50 ± 0.52	2.80 ± 0.94	7.23 ± 0.36
WM08	6.25 ± 0.00	18.75 ± 6.25	22.92 ± 3.61	0.53 ± 0.13	4.48 ± 1.61	9.44 ± 0.74
WM09	4.17 ± 3.61	12.50 ± 0.00	16.67 ± 3.61	0.36 ± 0.36	3.19 ± 0.92	7.77 ± 0.71
WM10	0	0	0	0	0	0
WM11	0	2.08 ± 3.61	4.17 ± 3.61	0	0.13 ± 0.22	1.07 ± 0.99
CICC33433	10.42 ± 3.61	22.92 ± 3.61	33.33 ± 3.61	1.94 ± 0.36	7.53 ± 1.04	15.31 ± 0.91
CICC33447	22.92 ± 3.61	39.58 ± 3.61	50.00 ± 6.25	3.81 ± 0.86	11.61 ± 2.25	21.63 ± 2.31
CICC32343	16.67 ± 3.61	33.33 ± 3.61	43.75 ± 6.25	3.18 ± 1.19	9.31 ± 1.11	18.60 ± 2.01
CICC1467	14.58 ± 3.61	33.33 ± 3.61	41.67 ± 7.22	2.99 ± 1.31	8.89 ± 2.18	18.53 ± 3.87
CGMCC2.3314	10.42 ± 3.61	29.17 ± 3.61	37.50 ± 6.25	1.70 ± 0.13	7.69 ± 0.42	14.77 ± 3.53

* DI% and LD (mm) is shown after 5 d, 7 d and 9 d (25 °C) of incubation. Each value is presented as the mean ± standard deviation.

Supplementary figure captions

Figure S1. Morphological characteristics of screened *Metschnikowia* yeasts on YPD+Fe medium. The symbols on the plates indicate the strain number. The concentration of $FeCl_3$ was 5 mg L^{-1} in the medium. Five strains of *M. pulcherrima* were used as controls: *M. pulcherrima* (CICC 33433, CICC 1467, CICC 32343 and CICC 33447) was purchased from the China Center of Industrial Culture Collection (CICC), and *M. pulcherrima* CGMCC 2.3314 was obtained from the China General Microbiological Culture Collection Center (CGMCC).

Figure S2. Effects of the *Metschnikowia* yeast isolates on the mycelial growth of *P. digitatum*, *P. italicum* and *G. citri-aurantii*. The symbols on the plates indicate the strain number.

Figure S1

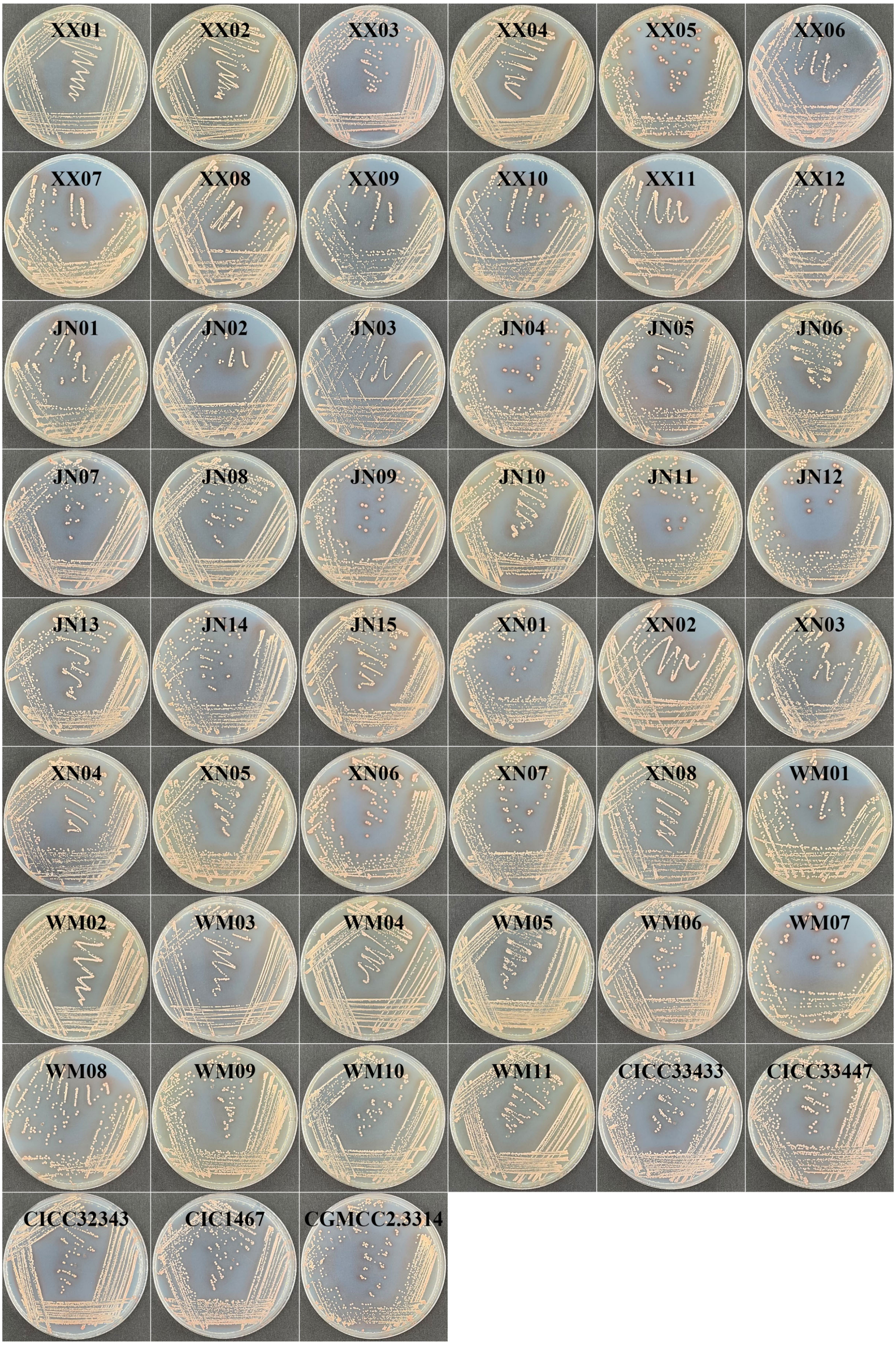
XX01
XX02
XX03
XX04
XX05
XX06
XX07
XX08
XX09
XX10
XX11
XX12
JN01
JN02
JN03
JN04
JN05
JN06
JN07
JN08
JN09
JN10
JN11
JN12
JN13
JN14
JN15
XN01
XN02
XN03
XN04
XN05
XN06
XN07
XN08
WM01
WM02
WM03
WM04
WM05
WM06
WM07
WM08
WM09
WM10
WM11
CICC33433
CICC33447
CICC32343
CIC1467
CGMCC2.3314

Figure S2

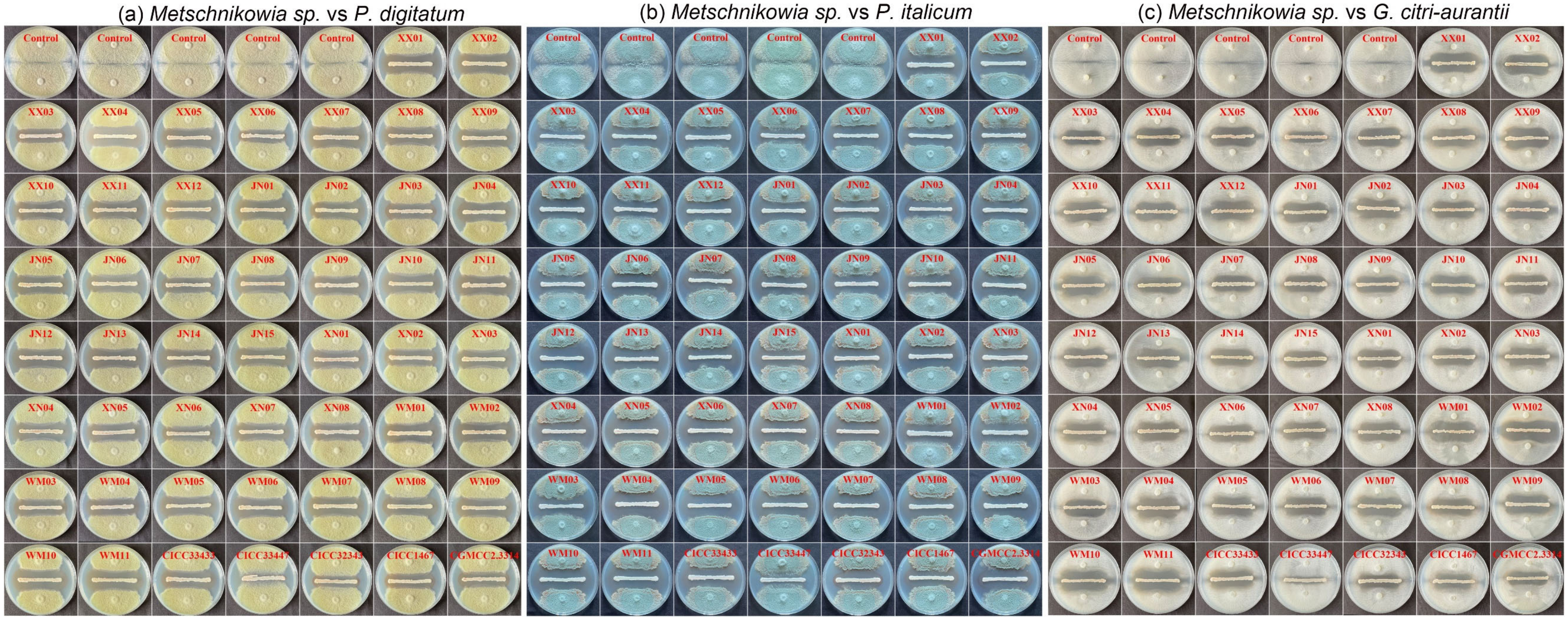
(a) *Metschnikowia sp.* vs *P. digitatum*
(b) *Metschnikowia sp.* vs *P. italicum*
(c) *Metschnikowia sp.* vs *G. citri-aurantii*
Control Control Control Control Control XX01 XX02
XX03 XX04 XX05 XX06 XX07 XX08 XX09
XX10 XX11 XX12 JN01 JN02 JN03 JN04
JN05 JN06 JN07 JN08 JN09 JN10 JN11
JN12 JN13 JN14 JN15 XN01 XN02 XN03
XN04 XN05 XN06 XN07 XN08 WM01 WM02
WM03 WM04 WM05 WM06 WM07 WM08 WM09
WM10 WM11 CICC33433 CICC33447 CICC32343 CICC1467 CGMCC2.3314
